# Staging of Endometrial Cancer Using Fusion T2-Weighted Images with Diffusion-Weighted Images: A Way to Avoid Gadolinium?

**DOI:** 10.3390/cancers14020384

**Published:** 2022-01-13

**Authors:** Teresa Resende Neves, Mariana Tomé Correia, Maria Ana Serrado, Mariana Horta, António Proença Caetano, Teresa Margarida Cunha

**Affiliations:** 1 Department of Radiology, Hospital Curry Cabral, Rua da Beneficência 8, 1050-099 Lisbon, Portugal; 71669@chlc.min-saude.pt; 2 Department of Radiology, Fundação Champalimaud, Avenida Brasília, 1400-038 Lisbon, Portugal; mariana.t.correia@fundacaochampalimaud.pt; 3 Department of Radiology, Hospital Central do Funchal, SESARAM, Avenida Luís de Camões, nº57, 9004-514 Funchal, Portugal; mariaanaserrado@sesaram.pt; 4 Department of Radiology, Instituto Português de Oncologia de Lisboa Francisco Gentil, Rua Professor Lima Basto, 1099-023 Lisbon, Portugal; mhorta@ipolisboa.min-saude.pt (M.H.); tmcunha@ipolisboa.min-saude.pt (T.M.C.); 5 Department of Radiology, Hospital da Luz Lisboa, Avenida Lusíada 100, 1500-650 Lisbon, Portugal; 71669@ chlc.min-saude.pt

**Keywords:** endometrial cancer, myometrial invasion, fused images, T2WI-DWI, accuracy

## Abstract

**Simple Summary:**

This work aims to evaluate the utility of fusing T2-weighted images with diffusion-weighted images to determinate the depth of myometrial invasion and the stage of endometrial cancer. By showing its superior diagnostic performance, we aim to encourage its use in endometrial cancer staging, and, in the future, obviate the need for intravenous contrast medium administration.

**Abstract:**

Endometrial cancer is the eighth most common cancer worldwide, and its prognosis depends on various factors, with myometrial invasion having a major impact on prognosis. Optimizing MRI protocols is essential, and it would be useful to improve the diagnostic accuracy without the need for other sequences. We conducted a retrospective, single-center study, which included a total of 87 patients with surgically confirmed primary endometrial cancer, and who had undergone a pre-operative pelvic MRI. All exams were read by an experienced radiologist dedicated to urogenital radiology, and the depth of myometrial invasion was evaluated using T2-Weighted Images (T2WI) and fused T2WI with Diffusion-Weighted Images (DWI). Both results were compared to histopathological evaluations. When comparing both sets of imaging (T2WI and fused T2WI-DWI images) in diagnosing myometrial invasion, the fused images had better accuracy, and this difference was statistically significant (*p* < 0.001). T2WI analysis correctly diagnosed 82.1% (70.6–88.7) of cases, compared to 92.1% correctly diagnosed cases with fused images (79.5–97.2). The addition of fused images to a standard MRI protocol improves the diagnostic accuracy of myometrial invasion depth, encouraging its use, since it does not require more acquisition time.

## 1. Introduction

According to the Global Cancer Observatory, endometrial cancer (EC) is the eighth most common cancer worldwide and the sixth most common cancer in female patients, with an incidence of 8.7 per 100,000 women per year [1,2]. The majority of cases are diagnosed at an early stage (around 80% in stage I), with a 5-year survival rate of 95% [3,4,5].

EC is classically divided into the following two histopathological types: type I (also known as endometrioid, estrogen-dependent), which comprises the majority of cases (80%) and is associated with a better outcome, and type II (nonendometrioid, nonestrogen-dependent; 20% of cases), which is associated with high-grade tumors and a dismal prognosis [4,6,7,8]. Endometrioid EC progresses from a premalignant phase, as an intraepithelial endometrial hyperplasia, while other histological types arise from various genetic mutations [6,9]. The identification of some gene mutations (i.e., loss of MSH2 or MLH1 genes) has become increasingly more important, given the emergence of immune checkpoint blockage therapies [6,8,10,11].

EC prognosis depends on several factors, and myometrial invasion is one of the most relevant findings, with a major impact on prognosis [12]. In fact, myometrial invasion is correlated with tumor grade, presence of lymph node metastasis, and overall survival [6,13,14]. More specifically, myometrial invasion beyond fifty percent of myometrium thickness is associated with a six to seven times increase in the prevalence of lymph node metastases [13,15,16].

Therefore, adequate evaluation of the precise local extension of EC is crucial to guide treatment [4]. Cross-sectional imaging techniques, in particular, magnetic resonance imaging (MRI), play a key role in the assessment of tumor extension and disease staging, and should be regarded as invaluable decision-making tools in such patients [4]. 

Each sequence has its own limitations that may sometimes make it challenging to correctly assess the extent of the disease [3,17]. Morphologic imaging is achieved with T2-Weighted Imaging (T2WI) sequences, but is generally combined with functional sequences (Diffusion-Weighted Imaging (DWI) and/or Dynamic Contrast Evaluation (DCE-MRI)) to establish the local staging of EC [18].

Although the accuracy of detecting EC myometrial invasion with morphologic and functional imaging is high, there is definitely room for improvement [3,13]. Recently, some authors have attempted to optimize the MRI protocol for EC evaluation by fusing T2WI with functional imaging [19,20]. This post-processing technique would allow for an improvement in diagnostic and staging accuracy, without the need to acquire other sequences that would otherwise increase the time and cost of the exam. The purpose of this study is to evaluate the additional diagnostic value of fusion T2WI-DWI images in staging EC, compared to the standard MRI evaluation.

## 2. Results

The population of this study had a median age of 68.8 years (ranging from 37 to 89 years old; N = 87). The surgical histopathological results are presented in Table 1. Endometrioid EC was diagnosed in 52 patients (59.8%), and, of the nonendometrioid type, mixed cell carcinoma was the most common (42.9%, *n* = 15), followed by serous EC (34.3%, *n* = 12).

Of the 87 patients studied, a total of 41 (47.1%) had proven deep myometrial invasion upon histopathologic evaluation, and, of those, 12 (29.0%) had serosal invasion and 24 (58.5%) had cervical stromal invasion.

The majority of patients were staged as FIGO IA (39.1%, *n* = 34), and, of the staged IVB patients, one had a proven spleen metastasis, which was previously detected by MRI, one had malignant cells in the peritoneal fluid, and five had peritoneal metastases at the time of surgery.

The percentage of cases diagnosed with superficial and deep myometrial invasion by standard MRI evaluation and fused T2WI-DWI was similar, namely, 55.2% vs. 56.3% for superficial invasion, and 44.8% vs. 43.7% for deep invasion, respectively (Table 2). Interestingly, fused images correctly diagnosed deep myometrial invasion in a total of 92.1% (79.5–97.2) cases, against 82.1% (69.4–90.2) correctly diagnosed with standard MRI sequences (Table 3). On other hand, fused images correctly excluded deep myometrial invasion in 87.8% (77.3–93.8) cases, against 81.2% (70.6–88.7) with standard MRI sequences (Table 3).

When comparing the accuracy of each set of imaging (standard MRI evaluation and fused T2WI-DWI images) in diagnosing deep myometrial invasion, the fused images showed better accuracy, and this difference was statistically significant (*p* < 0.001).

**Figure 1:**
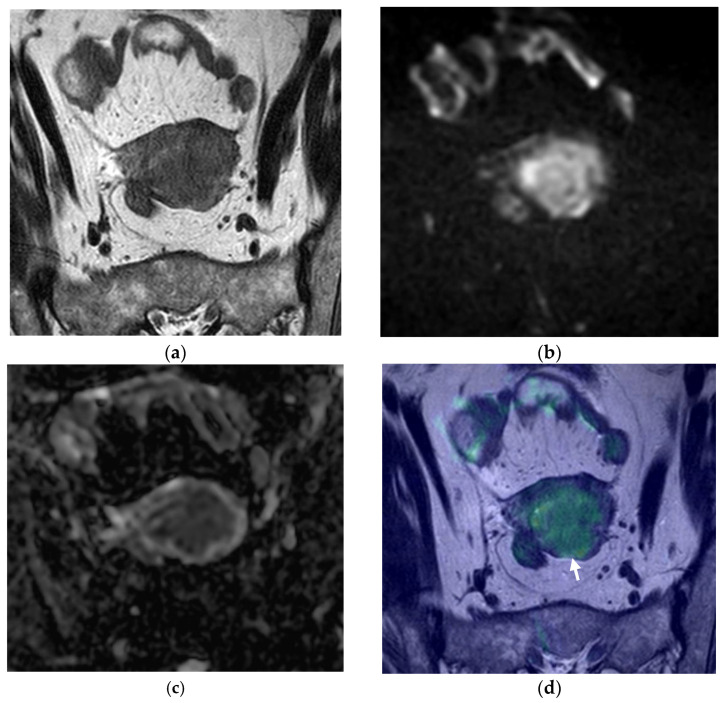
Pelvic MRI, axial oblique sequences oriented to the corpus uteri long axis: (**a**) T2WI; (**b**) DWI (b1000); (**c**) ADC map; (**d**) fused T2WI-DWI image. In (**a**), the endometrial cancer is hardly perceptible, being more depictable in functional images (**b**,**c**). It is difficult to establish if there is deep myometrial invasion using just the standard MRI sequences, but in analyzing the fusion image (**d**), it is clear that there is extension to the deep myometrial (arrow), which was confirmed with histopathological analysis.

**Figure 2:**
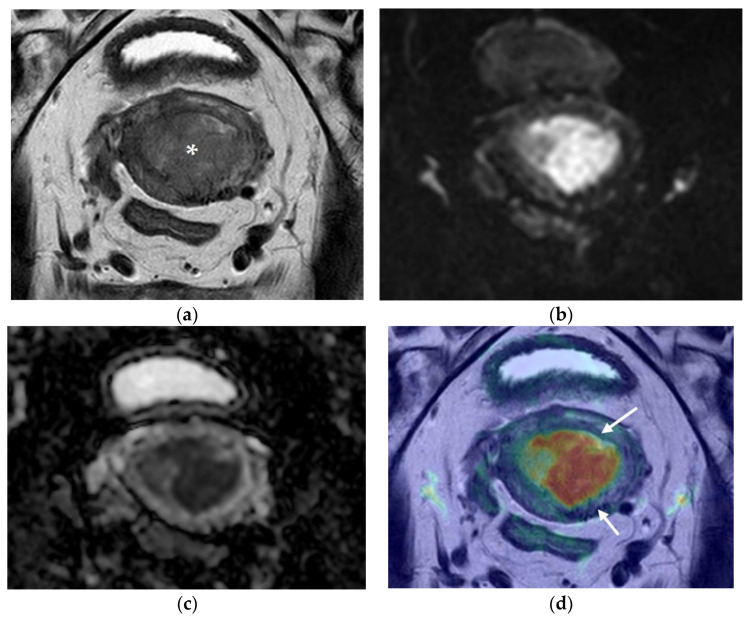
Pelvic MRI. Axial oblique sequences oriented to the corpus uteri long axis: (**a**) T2WI; (**b**) DWI (b1000); (**c**) ADC map; (**d**) fused T2WI-DWI image. Endometrial cancer is perceptible in (**a**) as a polypoid lesion with posterior implantation base (asterisk). When interpreting the morphologic (**a**) and functional images (**b**,**c**), deep myometrial invasion is questionable, but clearly excluded in fused images (**d**), where clear demarcation of the endometrial cancer and external myometrium is evident (arrows).

**Figure 3:**
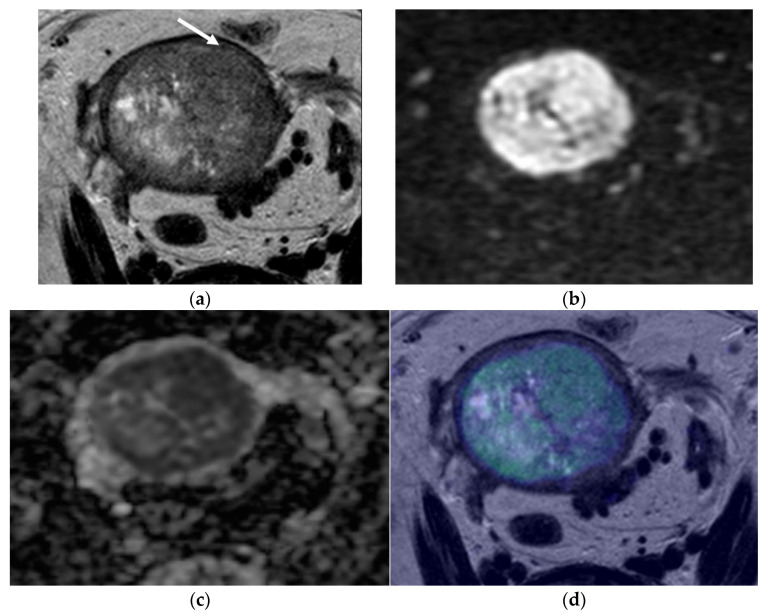
Pelvic MRI. Axial oblique sequences oriented to the *corpus uteri* long axis: (**a**) T2WI; (**b**) DWI (b1000); (**c**) ADC map; (**d**) fused T2WI-DWI image. In some cases, myometrial extension evaluation may be difficult, such as when the myometrium is thin, as is shown in this case. In these situations, fused images are of great value. In (**a**), an endometrial heterogeneous lesion is detected, markedly distending the endometrial cavity and compressing the myometrium (arrow), making it difficult to evaluate the depth of myometrial invasion. Fused images help to better demarcate the external myometrium and exclude invasion, which was suggested in (**d**) and confirmed by histopathological analysis.

Figure 1, Figure 2 and Figure 3 represent different scenarios where the addition of fused images led to a better determination of myometrial thickness and myometrial invasion by EC, and, in this matter, helped the diagnostician in regards to tumor staging.

## 3. Discussion

According to the results of this study, fused images (T2WI-DWI) have a statistically significantly higher capacity to predict the degree of myometrial invasion when compared to standard MRI evaluation with separate morphologic and functional sequences.

T2WI and DWI are well-established sequences used to determine the depth of myometrial invasion [18], and Gil et al. showed that adding DWI was superior to a morphologic T2WI protocol alone for the assessment of myometrial invasion and EC staging [3]. The authors also showed that an MRI protocol with DWI and T2WI performed slightly better than DCE combined with T2WI. There are, however, only a few studies evaluating the effectiveness of T2WI and DWI when fused together. 

A meta-analysis by Deng et al. demonstrated that the sensitivity of DWI combined or fused with T2WI, to evaluate myometrial invasion, was 85.8% (76.0–92.0), with a specificity of 94.7% (89.5–97.4) [13].

Guo et al. conducted a study aiming to investigate the performance of 3T-fused T2WI-DWI in assessing the depth of myometrial invasion and the staging of EC prior to surgery [19]. The authors demonstrated that fused T2WI-DWI images had higher diagnostic accuracy, sensitivity, specificity, and positive and negative predictive values compared to T2WI alone. Their work also showed that fused T2WI-DWI images exhibited higher diagnostic accuracy, for the assessment of tumor staging, compared to T2WI. Similarly, Shatat et al., despite having a small sample (N = 29), showed that fused images had higher diagnostic accuracy, sensitivity, specificity, and positive and negative predictive values compared to T2WI alone, for the evaluation of myometrial invasion. In both works, the authors demonstrated a sensitivity of 92.3% and 90%, respectively, and a specificity of 95.6% and 94.7%, respectively, for deep myometrial invasion [20]. These values are similar to what we obtained, with a higher specificity in the fused T2WI-DWI group in our series [93.5% (82.1–98.6); Table 3]. Table 4 presents the sensitivity, specificity, and accuracy values obtained in all three articles dedicated to this issue.

After careful evaluation of the misclassified cases, we have encountered some misleading cases already reported in the literature [7,17]. These are cases where the myometrium is thin (in postmenopausal women or in a polypod tumor markedly distending the endometrial cavity—Figure 3), cases where EC co-exists with leiomyomas or adenomyosis, which can cause distortion of the normal myometrium anatomy, and cases where the EC is isointense to the myometrium (Figure 1) [7,17]. With our work, we demonstrate that the addition of fused images helps to evaluate the depth of myometrial invasion.

Regarding the use of DCE, myometrial invasion is best depicted during the equilibrium phase (2 min 30 s after the injection) [18]. According to the recent metanalysis of Deng et al., DCE has a sensitivity of 86.3% (77.0–91.9) and a specificity of 86.5% (81.1–90.6) for evaluating myometrium invasion, with the specificity being inferior to what we found for fused images [93.5% (82.1–98.6)], and inferior to what is described for fused images in published articles on this subject [13,19,20]. As demonstrated in this metanalysis, and reported in other publications, these values are inferior to the sensitivity and specificity of the evaluation of T2WI combined with DWI [13,18]. Furthermore, there are cases where contrast administration is contraindicated. All that being said, we believe that the addition of fused images may have increased value for the correct evaluation of the depth of myometrial invasion.

Our work has some limitations. One limitation is related to the design, since it is a retrospective study. However, it should be noted that our radiological database presents accurate recordkeeping and a satisfactory temporal relationship between preoperative MRI, surgery, and histopathological reporting. The fact that all the exams were only read by a single radiologist might be considered a limitation to this work. However, the radiologist has more than 20 years of experience in urogenital radiology and is certified with the European Diploma in Urogenital Radiology (level III ESR European Training Curriculum for Subspecialization in Radiology). Another limitation was the absence of randomization in the analysis of standard MRI sequences, and then fused T2WI-DWI images (all morphological and DWI images were first evaluated, followed by all fused images). This could introduce some learning bias. However, within each group of studies analyzed (standard MRI evaluation versus fused T2WI-DWI evaluation), randomization was ensured. Furthermore, mismatch between the two sequences is a possibility, when performing image fusion, a limitation that may compromise the technique. Of the 87 MRIs that were read, we did not encounter this problem, maybe due to the scrupulous patient preparation and identical acquisition parameters employed for T2WI and DWI sequences across all patients.

## 4. Materials and Methods

### 4.1. Patient Selection

We conducted a retrospective, single-center study, which included a total of 87 patients. All patients had surgically confirmed primary EC and had undergone preoperative pelvic MRI. Patients were excluded if submitted to neoadjuvant treatment, had a previous history of pelvic neoplasia or were submitted to pelvic radiotherapy. This study was approved by the institutional review board and the requirement for written informed consent was waived due to its retrospective nature.

### 4.2. MRI Protocol

All MRI studies were performed in a 1.5 T MRI scanner (Intera Pulsar; Philips Medical Systems, Best, the Netherlands) with an 8-channel phased-array body coil and saturation bands (anterior and superior). A total of 4 h of fasting prior to examination was required, and patients were asked to empty both bladder and bowel before scanning. N-butylscopolamine bromide (20 mg) was administered intramuscularly, to reduce bowel motility and peristaltic artifacts. All patients were placed in the supine position.

Fast spin-echo T2WI (slice thickness, 6 mm; interslice gap, 1 mm; breath hold) and DWI with echo-planar technique (slice thickness, 6 mm; interslice gap, 1 mm; b-values of 0, 500, and 1000 s/mm^2^, together with the respective ADC maps) sequences were acquired from the diaphragm to the iliac crests for evaluation of advanced disease.

Pelvic evaluation included a fast spin-echo T1WI in the axial plane (slice thickness, 4 mm; interslice gap, 0.4 mm) and fast spin-echo T2WI acquired in the following three planes: axial (slice thickness, 4 mm; interslice gap, 0.4 mm), sagittal (slice thickness, 4 mm; interslice gap, 0.4 mm), and axial oblique of the uterine corpus (slice thickness, 4 mm; interslice gap, 0.4 mm). DWI with echo-planar technique was acquired in the axial plane (slice thickness, 4 mm; interslice gap, 1 mm; b-values of 0, 600, and 1000 s/mm^2^, with the respective ADC maps).

Fused images were obtained using the Multimodality Viewer application of the Phillips IntelliSpace Portal^®^. To avoid possible mismatch errors, both T2WI and DWI were obtained with the same parameters (orientation plane and slice thickness).

For the DCE sequence, a 3D-recalled echo fat-suppressed T1WI (slice thickness, 3 mm; interslice gap, 0.5 mm) was acquired before and after the intravenous injection of gadopentetate dimeglumine at a rate of 2 mL/s (0.1 mmol/kg of body weight—Magnevist; Bayer HealthCare AG, Leverkusen, Germany). Images were obtained in five different time periods (0, 25, 60, 120, and 150 s) in the axial oblique plane of the uterine corpus and at 240 s in the axial plane.

### 4.3. MRI Analysis

All exams were read by a radiologist specialized in urogenital radiology with more than 20 years of experience and with a European Diploma in Urogenital Radiology, blinded to the histopathological results.

The myometrial invasion depth was defined as the reason for the difference between the total thickness of normal myometrium in a non-invaded region and the thickness of the myometrium at the deepest point of tumor invasion, and was categorized as superficial if less than 50% of the myometrial thickness, and deep if higher than 50% of the myometrium thickness. 

All exam readings started with the analysis of morphological and functional images (T2WI and DWI, respectively) for determination of the depth of myometrial invasion and predictable stage of disease (considering the remaining parameters included in the staging system). Subsequently, fused T2WI-DWI images were analyzed according to the same parameters. In both readings, the T1WI images were evaluated independently to exclude potential pitfalls. Within each group of studies analyzed (combined morphological and functional evaluation or fused T2WI-DWI evaluation), randomization was ensured.

### 4.4. Histological Evaluation

The time lapse between the MRI and surgery varied between three to eight weeks, and all patients underwent total hysterectomy, bilateral salpingo-oophorectomy, pelvic lymphadenectomy, and peritoneal lavage. 

Every lesion was evaluated for myometrial invasion by an experienced pathologist specialized in female pelvic pathology, and was categorized in accordance with the 2018 FIGO staging system for postoperative pathologic staging [9].

### 4.5. Statistical Analysis

The software used for data analysis was IBM SPSS Statistics software, version 25 (IBM Corp., Armonk, NY, USA). Nominal variables were expressed as number and percentage (%). The sensitivity, specificity, and diagnostic accuracy of standard MRI evaluation and fused T2WI-DWI images were calculated and compared via the Pearson chi-square χ2 test. The accuracy of the different sets of sequences in the evaluation of deep myometrial invasion was analyzed through and compared with *p*-value adjustment by a logistic regression procedure. A *p*-value of <0.05 was regarded as statistically significant.

## 5. Conclusions

This study used a larger sample than those published in previous papers focused on this topic, and revealed a statistically significantly higher capacity of fused images in evaluating the degree of myometrial invasion compared to standard MRI evaluation with T2WI, DWI and DCE sequences. These results not only give strength to this technique, but also encourage its use, as it does not require more acquisition time and may obviate the use of contrast medium agents in the future. More studies are needed in this last topic, particularly when comparing fused images with DCE sequences.

## Figures and Tables

**Table 1:** Surgical histopathological results.

Variable	Percentage (*n*)
Histological Subtype	
Endometrioid	59.8 (52)
Nonendometrioid	40.2 (35)
Serous carcinoma	34.3 (12)
Clear cell carcinoma	11.4 (4)
Mixed cell carcinoma	42.9 (15)
Carcinossarcoma	5.7 (2)
Non differentiated	5.7 (2)
Tumor Grade	
1	28.1 (16)
2	38.6 (22)
3	33.3 (19)
Myometrial invasion	
Superficial (<50%)	52.9 (46)
Deep (≥50%)	47.1 (41)
2018 FIGO staging	
IA	39.1 (34)
IB	10.3 (9)
II	9.2 (8)
III	33.3 (29)
IVB	8.0 (7)

**Table 2:** MRI evaluation of endometrial invasion.

MRI Sequence	Superficial Myometrial Invasion Percentage (*n*)	Deep Myometrial Invasion Percentage (*n*)
Standard MRI evaluation	55.2 (48)	44.8 (39)
Fused T2WI-DWI	56.3 (49)	43.7 (38)

**Table 3:** Diagnostic performance of the standard MRI evaluation and fused T2WI-DWI images in evaluating the degree of myometrial invasion.

	Accuracy	Sensitivity	Specificity	Positive Predictive Value	Negative Predictive Value
Standard MRI evaluation % (95% CI)	81.6	78.1	84.5	82.1	81.2
	(71.6–89.1)	(62.4–89.4)	(71.1–93.7)	(69.4–90.2)	(70.6–88.7)
Fused T2WI-DWI % (95% CI)	89.7	85.4	93.5	92.1	87.8
	(81.3–95.2)	(70.8–94.4)	(82.1–98.6)	(79.5–97.2)	(77.3–93.8)

CI = confidence interval.

**Table 4:** Diagnostic performance of fused images in evaluating myometrial invasion.

Study	Sensitivity % (95% CI)	Specificity % (95% CI)	Accuracy
Guo et al.	92.3	95.6	94.8
Shatat et al.	90	94.7	93.1
Our results	85.4	93.5	89.7
	(70.8–94.4)	(82.1–98.6)	(81.3–95.2)

## Data Availability

The data presented in this study are available on request from the corresponding author.
